# Integrated single-cell and spatial transcriptomics combined with whole-exome sequencing reveal key hub genes and epithelial heterogeneity in bladder cancer

**DOI:** 10.3389/fonc.2025.1748105

**Published:** 2026-01-26

**Authors:** Lin Li, Qianyue Li, Jiaxin Liu, Yifan Wang, Yanjun Ma, Lei Tang, Yawei Zhao

**Affiliations:** 1 Department of Medical College, Shihezi University, Shihezi, Xinjiang, China; 2 Department of Urology, Xinjiang Production and Construction Corps Hospital, Urumqi, China; 3 Xinjiang Medical University, Urumqi, China

**Keywords:** bladder cancer, cancer-associated epithelial cells, single-cell RNA sequencing, spatial transcriptomics, tumor microenvironment, tumor mutation burden

## Abstract

**Background:**

Bladder cancer (BLCA) is a malignant tumor originating from the urothelial lining, characterized by a complex tumor microenvironment (TME) and heterogeneous tumor mutation burden (TMB). Cancer-associated epithelial cells (EpiCs) exhibit substantial heterogeneity during BLCA initiation and progression. Therefore, elucidating the diversity and functional states of EpiCs is essential for improving future diagnostic and therapeutic strategies.

**Methods:**

We integrated multi-omics datasets, including 13 single-cell RNA-seq samples, 514 bulk transcriptome profiles, and 30 whole-exome sequencing (WES) samples, to comprehensively characterize EpiC subtypes. Nonlinear dimensional reduction (UMAP) and clustering analyses were performed to identify major epithelial subsets, followed by secondary clustering. TMB values calculated from self-generated WES data were incorporated into scAB and Ro/e algorithms to determine the TMB-associated epithelial subset, ultimately identifying the key cluster Epi14. Differentially expressed genes (DEGs) of Epi14 were analyzed, and CellChat was used to infer intercellular communication networks. CytoTRACE and Monocle2 were applied to assess stemness potential and differentiation trajectories. Random survival forest (RSF) combined with DEG analysis was used to identify hub genes. Immune infiltration, drug sensitivity, and functional pathway analyses were subsequently conducted. Spatial transcriptomics were deconvoluted using spacexr, CellChat, and PROGENy to map cellular composition, signaling activity, and pathway nodes. Finally, qPCR and Western blot assays were performed to validate hub gene expression in tumor versus adjacent tissues.

**Results:**

A total of 77,263 cells and 3,000 highly variable genes were included, yielding 32 annotated cell clusters. Secondary clustering combined with WES-derived TMB identified 14 epithelial subpopulations, among which Epi14 was confirmed as the key TMB-associated subset using the Ro/e algorithm. Integration of DEGs, RSF, and multi-cohort datasets revealed ABRACL and ARPC3 as the pivotal hub genes, from which a risk-score model was constructed. Notably, ABRACL expression showed a strong positive association with tumor TMB and exhibited pronounced enrichment in spatial transcriptomic tumor regions.

**Conclusion:**

By integrating multi-omics and spatial datasets, this study reveals the epithelial heterogeneity of BLCA and identifies ABRACL and ARPC3 as key TMB-associated hub genes within EpiCs. The established risk-score model and validated functional markers provide valuable insights for future mechanistic studies and potential clinical translation in BLCA.

## 1 Introduction

Bladder cancer (BLCA) is one of the most common malignant tumors of the urinary system and arises from the urothelial epithelial lining (1). According to the 2022 Global Cancer Statistics, both the incidence and mortality of BLCA have increased compared with the 2020 report (2, 3). Despite progress in diagnostic and therapeutic approaches, clinical outcomes remain unsatisfactory. Challenges including immunotherapy resistance and treatment-related adverse events continue to hinder therapeutic efficacy, largely due to the complexity and heterogeneity of the TME and TMB (4–7). Therefore, identifying key hub genes within cancer-associated epithelial cells (EpiCs) has become a priority for improving BLCA management.

Advances in single-cell RNA sequencing (scRNA-seq), spatial transcriptomics (ST), whole-exome sequencing (WES), and machine learning (ML) have provided unprecedented opportunities to characterize the TME, dissect TMB-related mechanisms, and discover critical molecular drivers in BLCA (8–13). The TME is a dynamic ecosystem consisting of immune cells, fibroblasts, endothelial cells, and extracellular matrix components, all of which interact with tumor cells and shape disease progression (14–17). BLCA originates from urothelial EpiCs, which display extensive heterogeneity following malignant transformation. These transformed EpiCs can remodel the extracellular matrix and secrete cytokines, thereby establishing complex interactions with immune and stromal cells in the TME (18). Increasing evidence suggests that EpiC heterogeneity plays a decisive role in tumor progression, immune escape, and therapeutic responsiveness (19–21).

TMB reflects the total number of somatic mutations and is strongly associated with neoantigen generation and clinical benefit from immune checkpoint inhibitors (ICIs) (22, 23). High TMB has been shown to predict better immunotherapy responses across multiple cancers, including BLCA, hepatocellular carcinoma, colorectal cancer, lung cancer, and melanoma (24, 25). However, TMB alone cannot reveal cell-type–specific transcriptional differences or spatial organization within tumor tissues. Although scRNA-seq enables high-resolution profiling of cellular heterogeneity, it lacks spatial context. ST overcomes this limitation by preserving tissue architecture and linking gene expression to spatial coordinates, thereby enabling more accurate characterization of epithelial–immune–stromal interactions in BLCA. Integration of WES-derived TMB with scRNA-seq and ST data across independent cohorts allows multidimensional analyses at the genomic, transcriptional, and spatial levels, enabling cross-validation of epithelial heterogeneity and TMB-associated features (26–29).

In this study, we integrated scRNA-seq, ST, WES, bulk RNA-seq, and ML approaches to comprehensively investigate the BLCA microenvironment and identify key regulatory genes. TMB values derived from WES were mapped onto epithelial subsets, leading to the identification of a distinct TMB-associated EpiC subset, Epi14. Random survival forest (RSF) analysis combined with Epi14-specific differentially expressed genes (DEGs) was used to identify the hub genes ABRACL and ARPC3, followed by extensive downstream analyses. By integrating multi-dimensional multi-omics datasets, our study provides a comprehensive characterization of EpiC heterogeneity in BLCA. Furthermore, by incorporating TMB with multi-layered datasets, we propose a more refined strategy for predicting therapeutic responses in BLCA, offering new insights into personalized treatment and the identification of novel therapeutic targets.

## 2 Materials and methods

### 2.1 Data collection

A total of 30 paired BLCA tumor and adjacent normal tissue samples were collected from patients in the Department of Urology, Xinjiang Production and Construction Corps Hospital between September 2024 and September 2025. Among these, 15 pairs were used for bulk RNA-seq and WES, while the remaining 15 pairs were immediately snap-frozen in liquid nitrogen for subsequent analyses. This study was approved by the Ethics Committee of Xinjiang Production and Construction Corps Hospital (Approval No. 202403401), and written informed consent was obtained from all participants. All procedures complied with the Declaration of Helsinki (2013 revision).

scRNA-seq data were obtained from the GEO database. The GSE222315 dataset included 13 samples with complete scRNA-seq profiles (4 normal and 9 tumor samples). Bulk RNA-seq data from GSE236932 (platform GPL24676) included 63 samples (25 normal and 38 tumor). ST data were downloaded from GSE246011, containing two BLCA samples with complete spatial transcriptomic information. In addition, raw RNA-seq data for 421 BLCA samples were retrieved from the TCGA database, including 19 normal and 402 tumor samples.

### 2.2 TMB calculation

Fifteen BLCA tumor and matched adjacent tissue samples were subjected to WES using the Agilent SureSelect Human All Exon V6 kit (target region ~38 Mb). After obtaining the raw data, somatic mutation analysis was performed. ANNOVAR was used to annotate the mutation files provided by the sequencing company. To avoid duplicate counting caused by multiple transcript annotations, a unique mutation identifier was generated for each site based on chromosome (Chr), position (Pos), reference allele (Ref), and alternative allele (Alt), followed by removal of duplicates. Nonsynonymous exonic mutations—including missense, nonsense, splicing, frameshift insertion/deletion, and nonframeshift insertion/deletion variants—were retained, while synonymous and noncoding mutations were excluded. TMB for each sample was calculated by dividing the total number of filtered nonsynonymous mutations by the size of the Agilent V6 capture region (38 Mb), and expressed as mut/Mb. Given that BLCA generally exhibits a lower TMB level compared with cancers such as melanoma and lung cancer, and to avoid severe imbalance between groups in small- to moderate-sized cohorts, a fixed threshold of 10 mut/Mb was not used. Instead, samples were stratified into TMB-high and TMB-low groups based on the median TMB value for downstream analyses.

### 2.3 scRNA-seq quality control and cell annotation

scRNA-seq data were processed in R using the Seurat package. After importing the expression matrix, cells were filtered based on UMI counts, detected gene numbers, and mitochondrial gene percentages. Outliers were defined as cells deviating more than 3 median absolute deviations (MADs) from the median. According to the MAD distribution, cells with 200–4612 detected genes, <13026.67 UMIs, and <4.85% mitochondrial content were retained for downstream analysis.

Data were normalized using the LogNormalize method by scaling each cell to 10,000 UMIs followed by log-transformation. CellCycleScoring was applied to calculate cell cycle scores, FindVariableFeatures was used to identify highly variable genes, and ScaleData was performed to regress out variation associated with mitochondrial and ribosomal gene proportions as well as cell cycle effects. Dimensionality reduction was conducted using RunPCA, and batch effects were corrected using Harmony. Nonlinear reduction was then performed using RunUMAP to generate UMAP embeddings.

Cell type annotation was based on published markers and the CellMarker database, supplemented by automated predictions from SingleR. Manual curation was performed to finalize cluster identities and assign representative marker genes to each cell population.

### 2.4 Identification of TMB-associated epithelial cells using scAB and Ro/e

After cell annotation, epithelial cells were extracted for secondary analysis. These cells were processed in Seurat for normalization and scaling, followed by PCA and UMAP to obtain refined epithelial subclusters. TMB values calculated from WES for the 15 patient samples were stratified into TMB-high and TMB-low groups based on the median. The scAB (single-cell Association with Bulk phenotype) algorithm was then applied to map sample-level TMB phenotypes onto individual cells, enabling the identification of cell populations significantly associated with TMB status. scAB computed a phenotype-association probability for each cell, which reflects the degree to which an individual cell’s transcriptional profile aligns with the bulk-level gene expression patterns associated with the TMB-high phenotype. Biologically, higher scAB probabilities indicate epithelial cells whose transcriptional states are more strongly associated with high TMB status.

To quantify enrichment across epithelial subclusters, the Ro/e method was used to compare the observed number of scAB-identified cells in each subcluster with the expected number under a null distribution assuming random cell allocation. An Ro/e value greater than 1 indicates enrichment beyond random expectation, thereby supporting the identification of epithelial subclusters preferentially associated with the TMB phenotype. Through this combined scAB probability assessment and Ro/e enrichment analysis, the Epi14 subcluster was identified as an epithelial population significantly associated with the TMB-high group.

### 2.5 Identification of differentially expressed genes and enrichment analysis

Genes significantly associated with the Epi14 subcluster were extracted, and differential expression analysis was performed between scAB cells and Other cells. DEGs were identified using the criteria of log2FC > 0.585 and adjusted P < 0.05. The resulting DEGs were then subjected to functional enrichment analysis using the Metascape database (https://www.metascape.org), which included Gene Ontology (GO) functional classification and KEGG pathway analysis. These analyses were used to systematically explore the potential biological functions and signaling pathways associated with the identified DEGs.

### 2.6 Ligand - receptor interaction analysis, CytoTRACE - based stemness inference, and pseudotime trajectory reconstruction

To investigate the communication features and differentiation states of epithelial cells under different TMB levels, epithelial cells were grouped into TMB-Epi and Other epithelial cells. The CellChat package was used to analyze ligand–receptor interaction networks between the two groups and to identify potential signaling pathways and enriched communication patterns. CytoTRACE was applied to infer cellular stemness and differentiation potential based on transcriptional complexity, allowing estimation of developmental maturity and differentiation direction. To further characterize dynamic differentiation trajectories, pseudotime analysis was performed using Monocle2 to reconstruct developmental lineages of epithelial cells. Changes in key gene expression along the trajectory were examined to reveal temporal trends and potential regulatory roles during epithelial cell differentiation.

### 2.7 Identification of hub genes using machine learning and WES

A machine learning approach based on the random survival forest (RSF) algorithm was employed to identify prognostically relevant genes. Using the randomForestSRC package, RSF was performed on the previously identified DEGs, and genes with relative importance > 0.2 were selected as final feature genes. The selected candidate genes were subsequently subjected to survival analysis using overall survival (OS) data from the TCGA-BLCA cohort. OS was defined as the time from initial diagnosis to death from any cause or the last follow-up. Patients without observed events were censored at the date of last follow-up. The expression patterns of these genes were validated across the self-generated dataset, TCGA cohort, and GSE236932 dataset by comparing tumor versus adjacent normal tissues. Subsequently, WES data were used to further evaluate the association between candidate genes and TMB, enabling the identification of hub genes most closely related to the TMB status of tumor cells.

### 2.8 Construction of the nomogram model and drug sensitivity analysis

Based on the clinical data of BLCA patients, samples with missing information were excluded. An exploratory nomogram model was constructed using regression analysis to estimate 1-, 3-, and 5-year overall survival (OS). The model’s discriminative performance and potential clinical utility were internally evaluated using receiver operating characteristic (ROC) curves and decision curve analysis (DCA). For drug sensitivity assessment, the oncoPredict package combined with the regression framework was used to estimate the half-maximal inhibitory concentration (IC50) of commonly used chemotherapeutic agents in BLCA, thereby evaluating the sensitivity associated with the identified hub genes. The GDSC (Genomics of Drug Sensitivity in Cancer) dataset was used as the training set, and 10-fold cross-validation was performed to ensure model robustness and predictive accuracy.

### 2.9 Immune infiltration analysis, GSEA, and GSVA

Using self-generated bulk RNA-seq data, immune cell fractions were estimated with the CIBERSORT algorithm to deconvolute the expression matrix into 22 immune cell subtypes. Pearson correlation analysis was performed to evaluate the relationships between hub gene expression levels and immune cell infiltration, thereby revealing potential interactions between key genes and the immune microenvironment. To explore signaling pathways associated with the hub genes, GSEA was conducted by comparing high- and low-expression groups to identify significantly enriched biological pathways. Additionally, Hallmark gene sets were obtained from the MSigDB database, and GSVA was applied to calculate enrichment scores for each gene set, enabling the evaluation of functional differences and pathway activity variation across samples.

### 2.10 Spatial transcriptomics analysis

Spatial transcriptomics (ST) data were processed through standard normalization, scaling, and PCA-based linear dimensionality reduction, followed by Bayesian clustering to identify spatially defined cell subpopulations. The spacexr package was applied to integrate ST data with scRNA-seq profiles for deconvolution, enabling estimation of the relative proportions of different cell types within each spatial spot. CellChat was used to analyze ligand–receptor interactions based on key feature genes identified in scRNA-seq data, allowing exploration of potential communication networks between TMB-Epi cells and other cell lineages. PROGENy was employed to construct pathway-specific core gene sets and calculate pathway activity scores, thereby pinpointing critical signaling nodes associated with the TMB-Epi subcluster within BLCA tissues. Finally, the spatial expression patterns of the identified hub genes were visualized and analyzed across the ST dataset.

### 2.11 PCR validation

Total RNA was extracted using the TRIzol reagent kit, and cDNA was synthesized using a reverse transcription kit. The resulting cDNA was mixed with SYBR Green RT-qPCR Master Mix, and RT-qPCR was performed on the CFX96™ real-time PCR system. β-actin served as the internal control, and mRNA expression levels were quantified using the 2^-ΔΔCt method. The primer sequences were as follows: β-actin-F: 5′-CACCATTGGCAATGAGCGGTTC-3′; β-actin-R: 5′-AGGTCTTTGCGGATGTCCACGT-3′; ARPC3-F: 5′-ACTCATCGGAAACATGGCACT-3′; RNASET2-R: 5′-TCTTTTGTCTCTCTGGGGGC-3′; ABRACL-F: 5′-ATTTGGGGTCCTCTTCCGTG-3′;ABRACL-R: 5′-TCTTCCTTCGTTTTGCAGCTTT-3′.

### 2.12 Western blot validation

Approximately 10 mg of each tissue sample was homogenized in lysis buffer containing RIPA, PMSF, and a protease inhibitor cocktail (volume ratio 100:1:1). After thorough vortexing and centrifugation, the supernatant was collected, and protein concentration was quantified using the BCA assay. Protein samples were mixed with 5 × loading buffer at a 1:4 ratio and denatured at 100°C for 10 min. Proteins were separated by SDS-PAGE and transferred onto PVDF membranes. Membranes were blocked using a rapid blocking solution and washed with TBST containing Tween. Primary antibodies against ABRACL, ARPC3, and β-actin were incubated with the membranes overnight at 4°C. After TBST washing, membranes were incubated with HRP-conjugated secondary antibodies for 1 h at room temperature. Protein signals were detected using an enhanced chemiluminescence (ECL) imaging system. β-actin served as the internal loading control. The expression levels of ABRACL and ARPC3 in tumor and adjacent tissues were quantified based on grayscale intensity.

### 2.13 Statistical analysis

Unless otherwise specified, all statistical analyses and data visualization were performed using R software (version 4.5.1) and the corresponding R packages. A two-tailed P < 0.05 was considered statistically significant.

## 3 Result

### 3.1 scRNA-seq reveals major cellular clusters in BLCA

**Figure 1:**
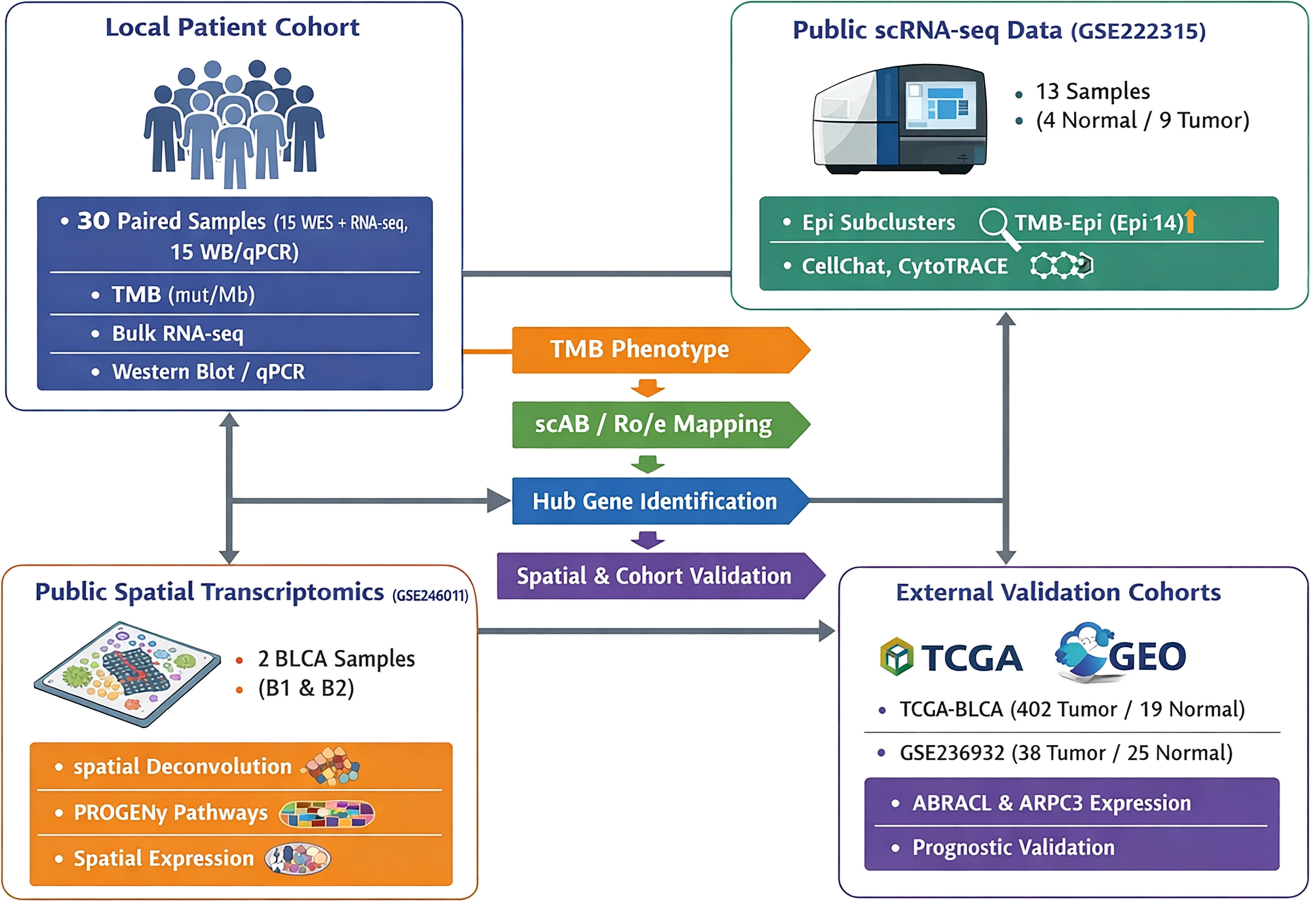
The schematic diagram of this study.

**Figure 2:**
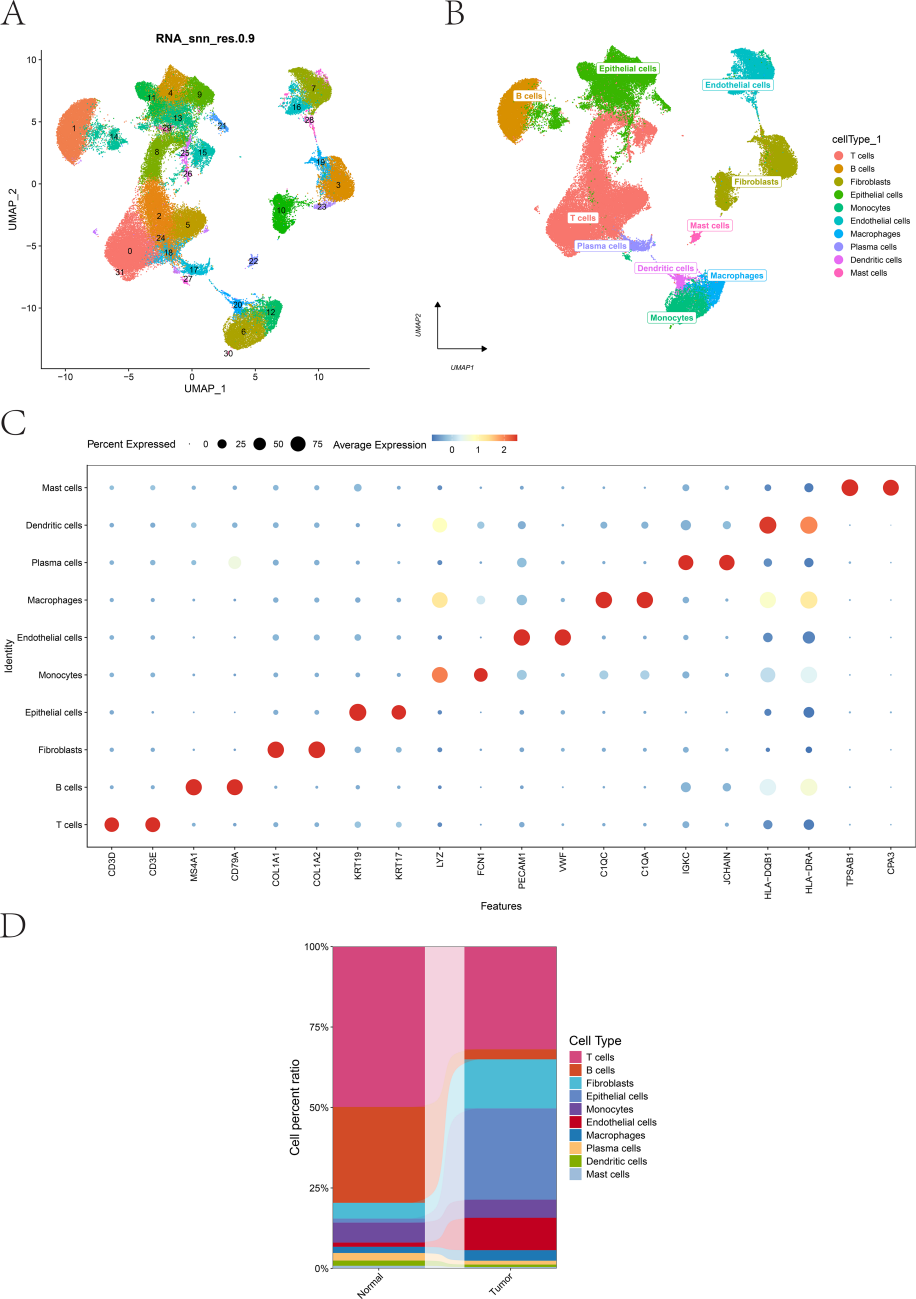
Single-cell landscape of bladder cancer. **(A)** UMAP visualization of scRNA-seq data showing the clustering distribution of bladder cancer cells. **(B)** UMAP plot displaying annotated cell types across all clusters. **(C)** Feature plots showing the expression patterns of representative marker genes for each major cell type. **(D)** Bar plot comparing the proportions of major cell lineages between normal bladder tissues and BLCA tumor tissues.

A schematic illustration of the study design is presented in the figure (**Figure 1**). A total of 13 BLCA-related scRNA-seq samples were included, and 77,263 cells were retained after quality control. Following data normalization, scaling, PCA, Harmony batch correction, and UMAP dimensionality reduction, 32 distinct clusters were identified (**Figure 2A**). These clusters were annotated into 10 major cell types, including T cells, B cells, fibroblasts, epithelial cells, monocytes, endothelial cells, macrophages, plasma cells, dendritic cells, and mast cells (**Figure 2B**). Representative marker gene expression patterns for these cell types are shown in (**Figure 2C**) and the proportions of the 10 cell lineages in normal and tumor tissues are illustrated in (**Figure 2D**).

### 3.2 Re-clustering of EpiCs and identification of TMB-associated DEGs

**Figure 3:**
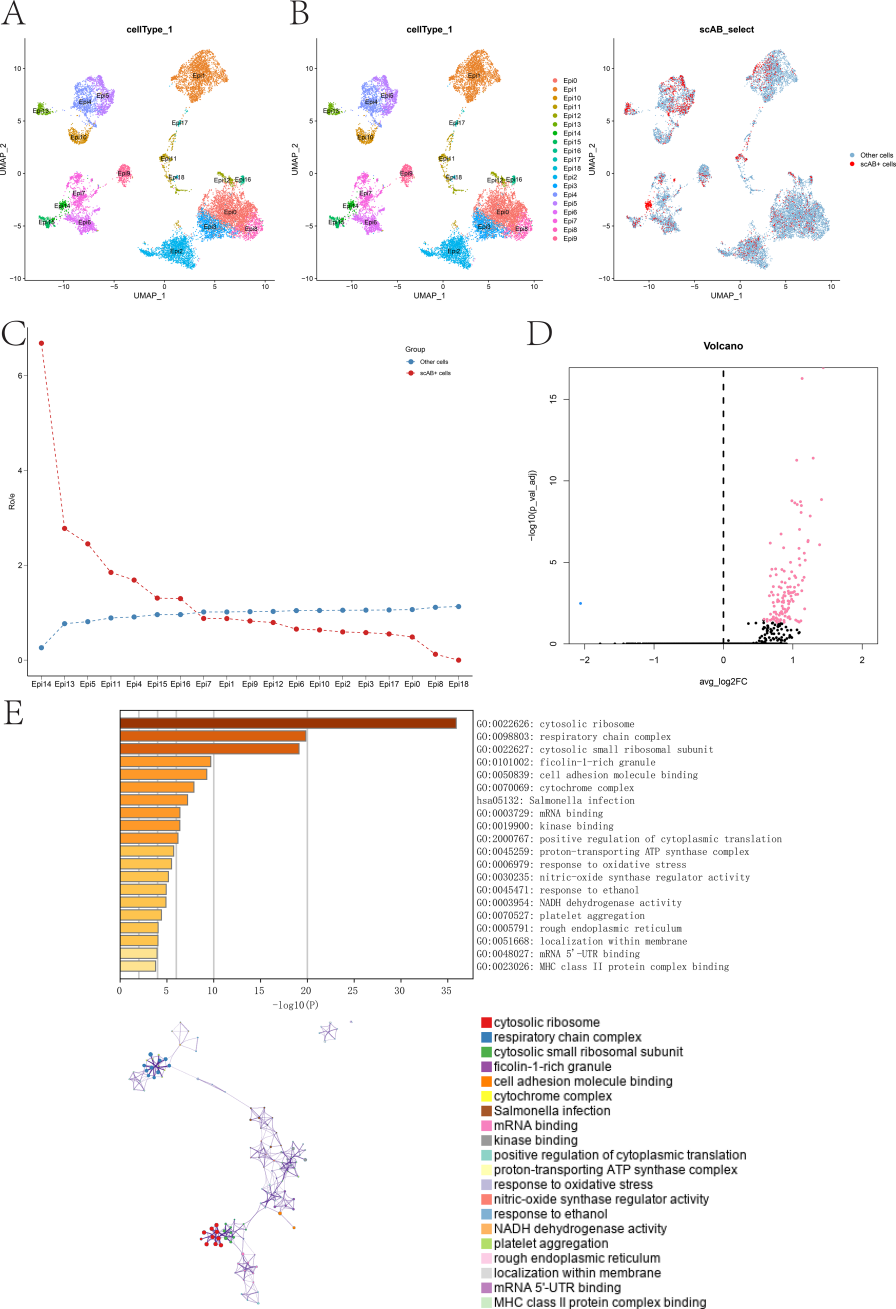
Identification of TMB-associated epithelial subclusters. **(A)** Secondary clustering and UMAP visualization of epithelial cells (EpiCs), identifying 19 subclusters. **(B)** Distribution of scAB cells showing predominant enrichment within the Epi14 subcluster. **(C)** Observed-to-expected (Ro/e) enrichment comparison between scAB cells and Other cells across epithelial subclusters. **(D)** Volcano plot showing differentially expressed genes (DEGs) between scAB cells and Other cells. **(E)** Functional enrichment analysis of DEGs using Metascape.

EpiCs were isolated and subjected to secondary clustering, resulting in the identification of 3,000 highly variable genes. After secondary dimensionality reduction, 19 epithelial subclusters (Epi0–Epi18) were obtained (**Figure 3A**). Using the scAB algorithm combined with TMB values, cells significantly associated with high TMB were identified and designated as scAB cells (**Figure 3B**). Ro/e ratio analysis demonstrated that scAB cells were predominantly enriched within the Epi14 subcluster, whereas Other cells were observed at lower-than-expected frequencies (**Figure 3C**), thus establishing Epi14 as the key TMB-associated epithelial subpopulation. Genes specifically associated with Epi14 were extracted, and differential expression analysis between scAB cells and Other cells identified 119 significant DEGs (|log_2_FC| > 0.585, FDR < 0.05) (**Figure 3D**). Metascape enrichment analysis revealed that these DEGs were mainly enriched in pathways related to the cytosolic ribosome and respiratory chain complex (**Figure 3E**).

### 3.3 Ligand–receptor interactions, cellular differentiation status, and pseudotime trajectory

**Figure 4:**
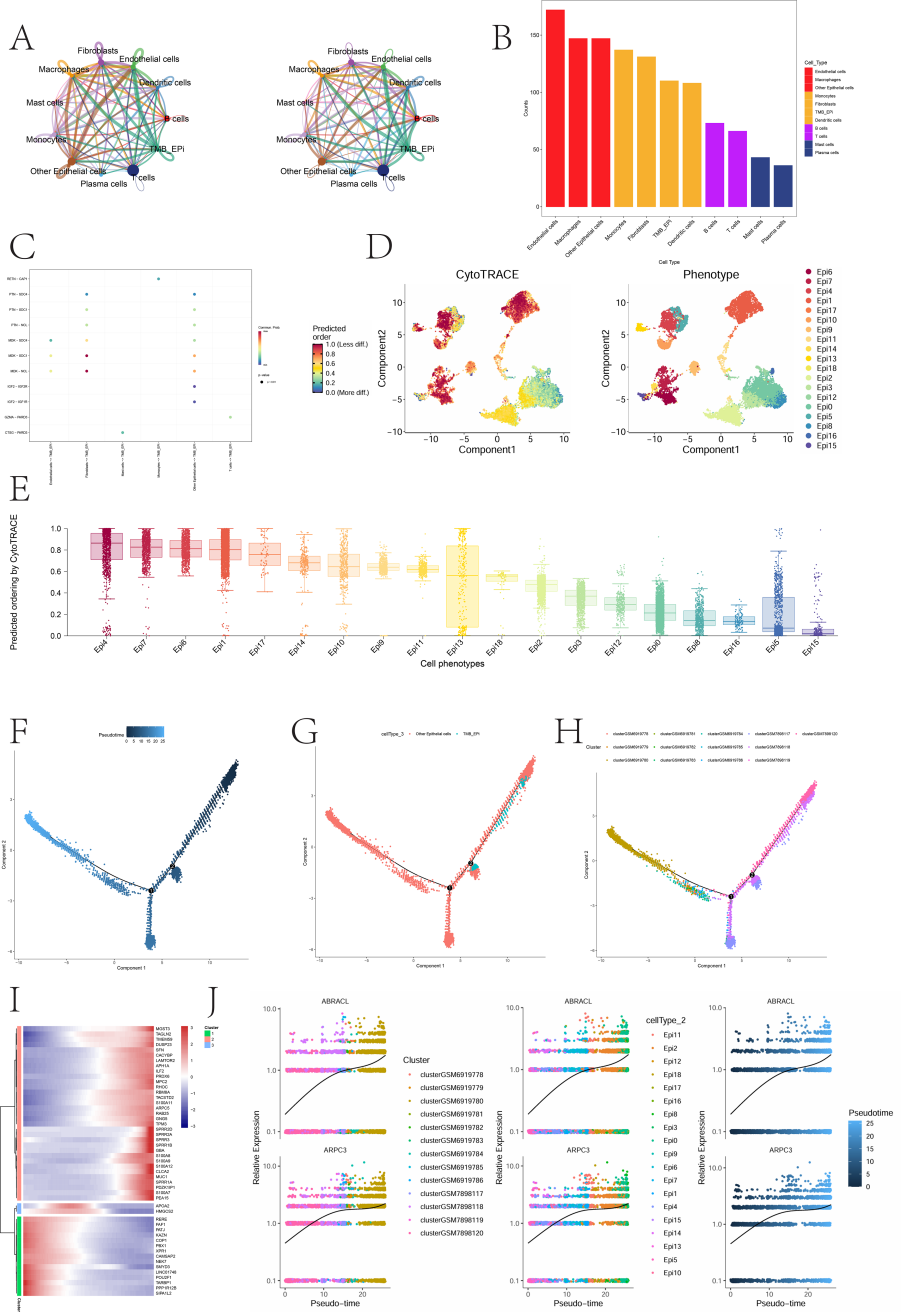
Cell–cell communication, differentiation potential, and pseudotime dynamics of epithelial subclusters. **(A, B)** CellChat analysis showing interaction networks between TMB-Epi cells and other cell types. **(C)** Communication patterns from non-epithelial cells toward TMB-Epi cells. **(D, E)** CytoTRACE analysis indicating stemness gradients and differentiation potential across EpiC subclusters. **(F-H)** Pseudotime trajectory reconstruction demonstrating that TMB-Epi cells are primarily positioned at early differentiation stages. **(I, J)** Heatmap of dynamic gene expression changes along pseudotime and expression trends of ABRACL and ARPC3 throughout the differentiation trajectory.

Cell–cell communication analysis using CellChat revealed extensive signaling networks across different cell populations within BLCA (**Figure 4A**). Notably, interactions between TMB-Epi cells and endothelial cells, macrophages, and fibroblasts were among the strongest in both number and intensity (**Figure 4B**). Further analysis identified the MDK–SDC1 and MDK–NCL signaling axes as key communication routes from fibroblasts to TMB-Epi cells (**Figure 4C**), suggesting potentially important regulatory roles in epithelial cell behavior. CytoTRACE analysis was performed to estimate the stemness and differentiation potential of EpiC subpopulations. A gradual decline in stemness was observed from Epi4 to Epi15 (**Figures 4D, E**), with Epi14 exhibiting an intermediate-to-high stemness state, consistent with its strong association with elevated TMB. To further characterize developmental dynamics, pseudotime reconstruction using Monocle2 was conducted (**Figures 4F–H**). TMB-Epi cells were predominantly positioned at the early stages of the trajectory, indicating a relatively primitive differentiation status. Gene expression dynamics along the pseudotime trajectory (**Figures 4I, J**) showed that key genes such as ABRACL and ARPC3 displayed an increasing trend during differentiation. These genes are associated with cellular migration and differentiation processes, indicating their potential involvement in TMB-associated functional reprogramming of epithelial cells.

### 3.4 Identification and validation of ABRACL and ARPC3 as hub genes through machine learning and multi-dataset integration

**Figure 5:**
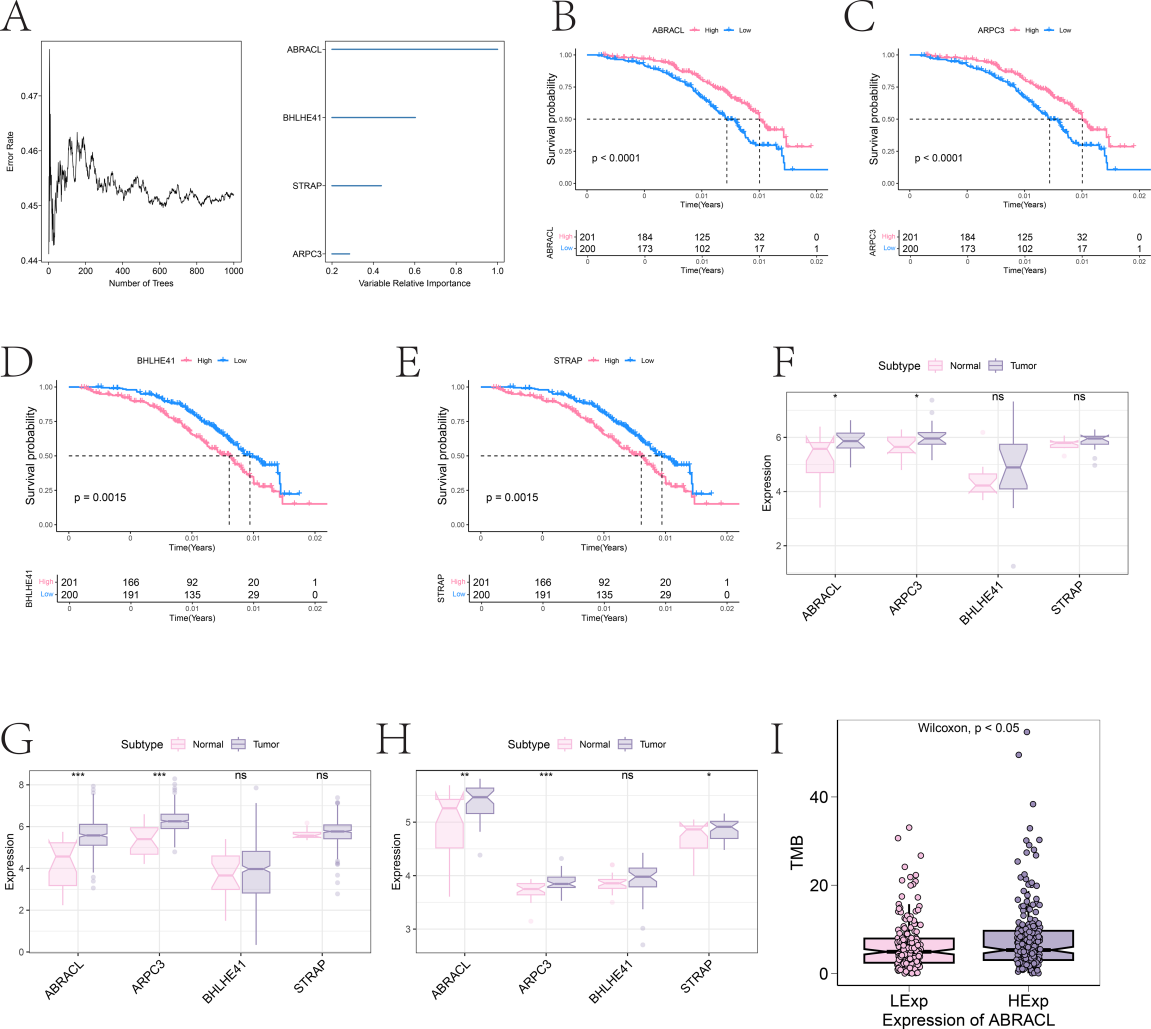
Identification and validation of key TMB-associated genes. **(A)** Error rate curve of the random survival forest (RSF) model and selection of key genes based on relative importance. **(B–E)** Kaplan–Meier survival curves for ABRACL, ARPC3, BHLHE41, and STRAP, showing their associations with overall survival. **(F–H)** Differential expression of the four candidate genes between normal and tumor tissues across the self-generated dataset, TCGA cohort, and GSE236932 dataset. **(I)** Association between expression levels of key genes and TMB values. *, **, and *** indicate statistical significance levels of P < 0.05, P < 0.01, and P < 0.001, respectively.

Using the RSF algorithm for feature selection, genes with a relative importance > 0.2 were retained, resulting in the identification of four candidate genes: ABRACL, ARPC3, BHLHE41, and STRAP (**Figure 5A**). Survival analysis demonstrated that the expression levels of all four genes were significantly associated with overall survival (P < 0.05) (**Figures 5B–E**). Expression validation using the self-generated cohort, TCGA dataset, and GSE236932 dataset showed that ABRACL and ARPC3 were consistently upregulated in tumor tissues, whereas BHLHE41 and STRAP did not exhibit consistent differential expression across datasets (**Figures 5F–H**). Further analysis incorporating WES-derived TMB values revealed that samples with high ABRACL expression displayed markedly elevated TMB levels (**Figure 5I**), whereas the low-expression group showed substantially reduced TMB. These findings suggest that ABRACL overexpression may be closely related to increased mutational burden in tumor cells and may play a pro-tumorigenic role in BLCA progression.

### 3.5 Immune infiltration, GSEA, and GSVA analyses

**Figure 6:**
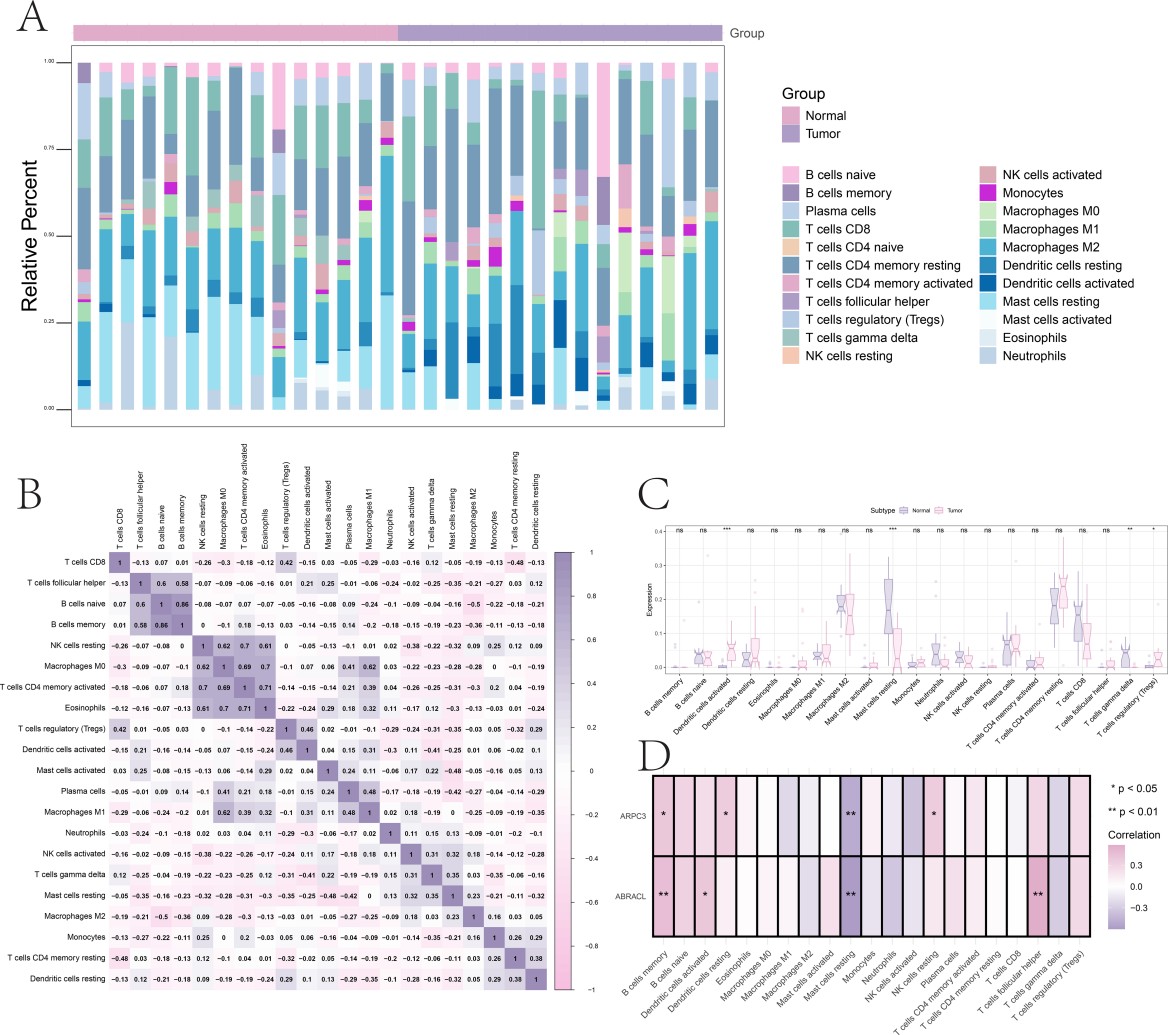
Immune infiltration landscape and correlation with hub genes. **(A)** Differences in the proportions of 22 immune cell subtypes between groups. **(B)** Pearson correlation heatmap showing interrelationships among immune cell populations. **(C, D)** Correlation heatmaps illustrating the associations between hub genes (ABRACL and ARPC3) and immune cell abundances. *, **, and *** indicate statistical significance levels of P < 0.05, P < 0.01, and P < 0.001, respectively.

Using self-generated bulk RNA-seq data, CIBERSORT was applied to estimate the relative abundance of 22 immune cell subtypes. Significant differences in immune infiltration profiles were observed between the Normal and Tumor groups (**Figure 6A**). Among these, activated dendritic cells, resting mast cells, γδ T cells, and regulatory T cells (Tregs) showed the most pronounced differences (Wilcoxon test, P < 0.05). Correlation heatmaps further revealed complex cooperative and antagonistic relationships among immune cell populations (**Figure 6B**).

We next examined the associations between hub genes and immune cell infiltration. ABRACL expression was positively correlated with memory B cells, follicular helper T cells, and activated dendritic cells, but negatively correlated with resting mast cells. ARPC3 expression showed positive correlations with memory B cells, resting NK cells, and resting dendritic cells, while also displaying a negative association with resting mast cells (**Figures 6C, D**).

**Figure 7:**
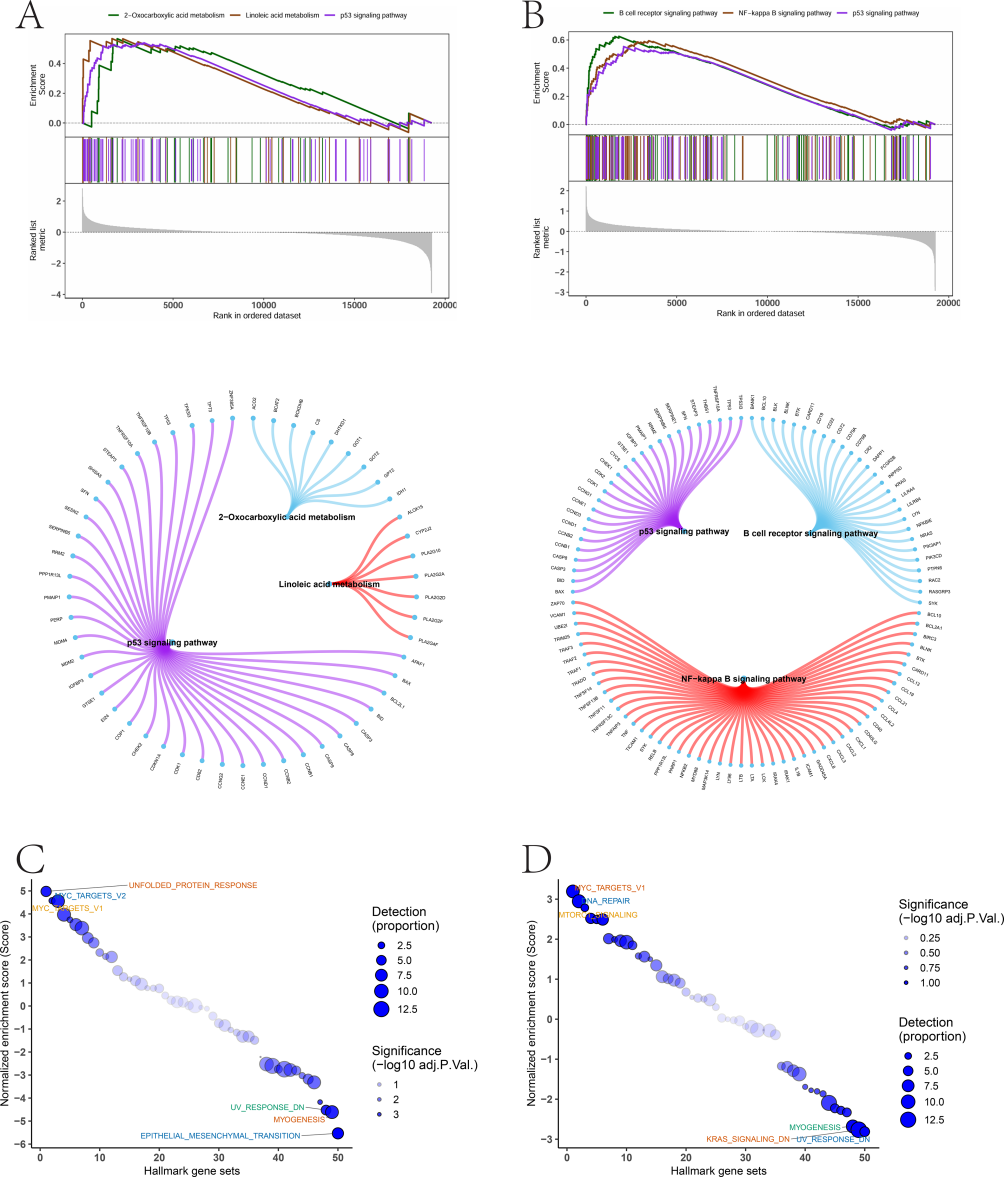
Pathway enrichment and functional activity associated with hub genes. **(A, B)** GSEA identifying significantly enriched signaling pathways associated with differential expression patterns. **(C, D)** GSVA showing pathway activity scores linked to ABRACL- and ARPC3-related functional programs.

GSEA revealed that ABRACL was enriched in pathways such as the p53 signaling pathway, 2-oxocarboxylic acid metabolism, and linoleic acid metabolism. ARPC3 was enriched in pathways including the B cell receptor signaling pathway, NF-κB signaling pathway, and p53 signaling pathway (**Figures 7A, B**). GSVA analysis further indicated that ABRACL was enriched in UNFOLDED PROTEIN RESPONSE and MYC TARGETS V2 signatures, whereas ARPC3 showed enrichment in MYC TARGETS V1 and DNA REPAIR pathways (**Figures 7C, D**).

### 3.6 Prognostic evaluation and drug sensitivity characteristics of hub genes

**Figure 8:**
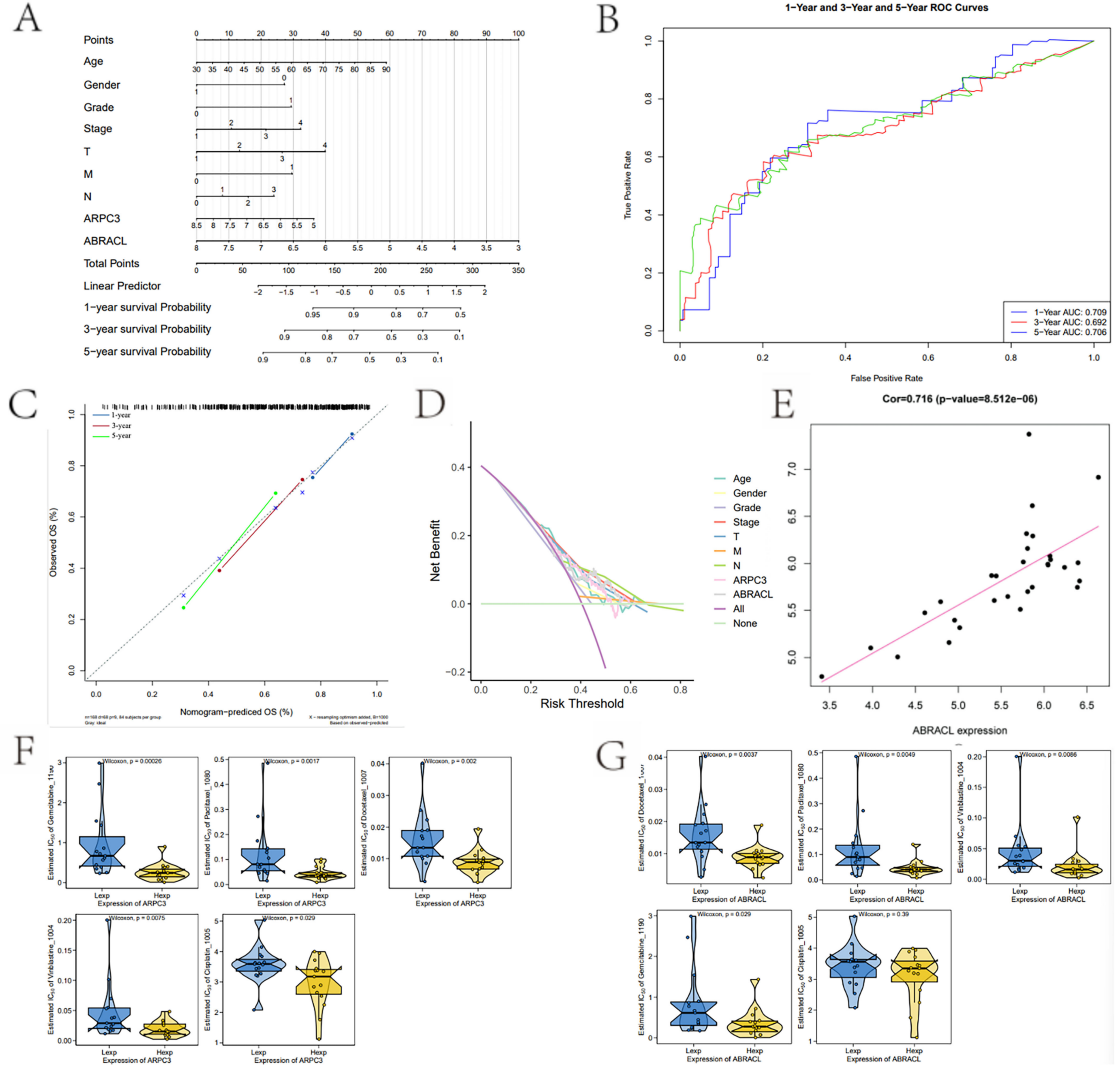
Exploratory prognostic model construction and drug sensitivity analysis of hub genes. **(A)** Exploratory nomogram integrating hub genes and clinical variables for predicting 1-, 3-, and 5-year overall survival. **(B)** ROC curves evaluating the predictive performance of the nomogram. **(C)** Calibration curves illustrating agreement between predicted and observed survival outcomes. **(D)** Decision curve analysis (DCA) demonstrating the clinical net benefit of the model across various threshold probabilities. **(E)** Correlation analysis showing the relationship between ABRACL and ARPC3 expression levels. **(F, G)** Drug sensitivity differences between high- and low-expression groups of hub genes, based on predicted IC50 values.

To evaluate the potential prognostic relevance of the hub genes, the expression levels of ABRACL and ARPC3 were integrated with clinical variables to construct an exploratory nomogram model for predicting 1-, 3-, and 5-year overall survival (OS) in BLCA patients (**Figure 8A**). The model demonstrated strong contributions from both genes to the overall risk score. Calibration curves indicated excellent agreement between predicted and observed survival outcomes (**Figure 8C**). ROC curve analysis further showed that the AUC values for 1-, 3-, and 5-year OS were all approximately 0.7 (**Figure 8B**), suggesting good discriminative performance. In addition, DCA confirmed that the nomogram provided higher net clinical benefit across a broad range of threshold probabilities (**Figure 8D**).

To explore the roles of these genes in chemotherapeutic sensitivity, IC50 values were predicted using GDSC drug data based on the oncoPredict algorithm. The high ARPC3 expression group exhibited significantly lower IC50 values than the low-expression group, indicating increased sensitivity to gemcitabine, cisplatin, and docetaxel (**Figure 8F**). Similarly, the high ABRACL expression group showed reduced IC50 values and enhanced sensitivity to gemcitabine, paclitaxel, and docetaxel (**Figure 8G**). Correlation analysis using self-generated expression data revealed a strong positive relationship between ARPC3 and ABRACL expression (Pearson r = 0.716, P = 8.5×10^-6^) (**Figure 8E**). These findings suggest that ABRACL and ARPC3 may jointly contribute to chemotherapy responsiveness in BLCA through a coordinated regulatory mechanism, with drug sensitivity results representing in silico predictions intended to guide future experimental validation.

### 3.7 Spatial cell–cell communication, pathway activity, and spatial expression of hub genes

**Figure 9:**
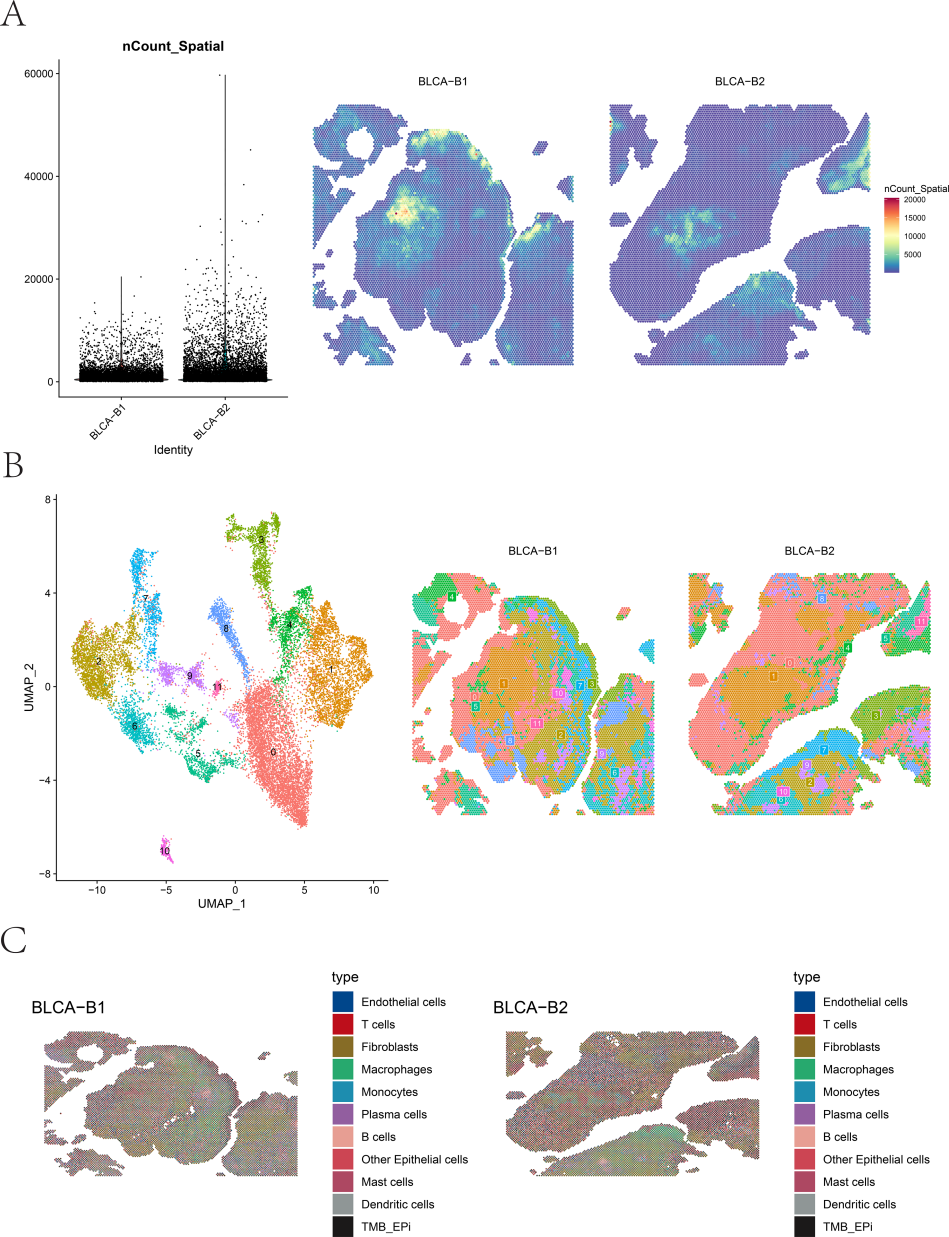
Spatial clustering and cell-type distribution in BLCA tissues. **(A, B)** Spatial clustering results and UMAP visualization of ST data from BLCA-B1 and BLCA-B2, including quality control metrics. **(C)** Spatial distribution of major cell types across BLCA-B1 and BLCA-B2 tissue sections, as inferred from deconvolution analysis.

To further investigate the spatial distribution patterns of distinct cell subtypes and their functional interactions within BLCA tissues, spatial transcriptomics datasets BLCA-B1 and BLCA-B2 were comprehensively analyzed. After data normalization, scaling, PCA dimensionality reduction, and Bayesian clustering, multiple spatially distinct clusters were identified within the tissue sections (**Figures 9A, B**). Spatial expression matrices (nCount_Spatial) revealed densely expressed regions in both BLCA-B1 and BLCA-B2, indicating transcriptionally active tumor areas. Deconvolution using the spacexr package, integrated with scRNA-seq data, was performed to estimate cell-type composition across spatial spots. The results showed that epithelial cells, fibroblasts, macrophages, and various immune populations were widely distributed, exhibiting marked spatial heterogeneity across different tissue regions (**Figure 9C**).

**Figure 10:**
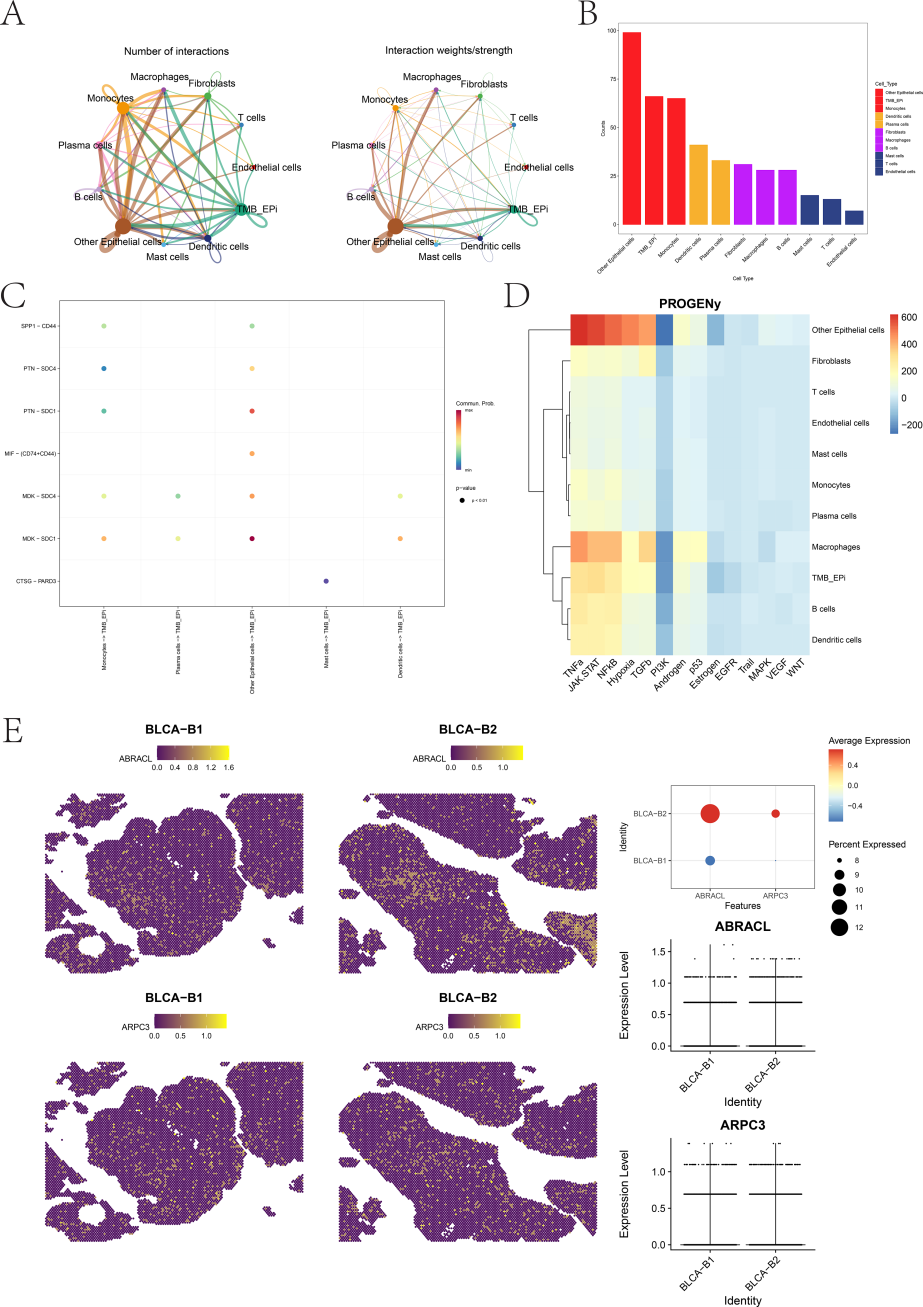
Cell–cell communication networks, pathway activity, and spatial expression of hub genes. **(A, B)** CellChat network analysis illustrating global intercellular communication patterns across BLCA cell populations. **(C)** Interaction network highlighting significantly enriched ligand–receptor pairs between TMB-Epi cells and neighboring stromal or immune cells. **(D)** Heatmap of pathway activity scores computed using PROGENy across major cell types. **(E)** Spatial expression patterns and quantitative comparison of ABRACL and ARPC3 in BLCA-B1 and BLCA-B2 spatial transcriptomics samples.

Next, ligand–receptor interaction analysis was conducted using CellChat based on scRNA-seq features to reconstruct the cellular communication network within BLCA tissues (**Figures 10A, B**). Substantial interactions were observed among multiple cellular populations, with TMB-Epi showing strongly enhanced communication strength and frequency with fibroblasts, endothelial cells, and macrophages. Notably, signaling axes such as MDK–SDC1, MDK–SDC4, and PTN–SDC1 were highly active between TMB-Epi and neighboring stromal cells (**Figure 10C**), suggesting their potential regulatory roles in tumor epithelial–stromal communication. Pathway activity analysis using PROGENy further demonstrated that the TMB-Epi subcluster exhibited markedly elevated JAK-STAT activity, accompanied by activation of TNFα and NF-κB pathways (**Figure 10D**), highlighting the inflammatory and pro-tumorigenic signaling features of this epithelial subtype. Finally, the spatial expression patterns of the hub genes ABRACL and ARPC3 were examined (**Figure 10E**). Both genes showed markedly higher expression in tumor-dense regions (BLCA-B2) compared to regions with lower tumor content (BLCA-B1), and their spatial distributions were highly concordant. These findings support their coordinated roles in regulating cytoskeletal remodeling and signal transduction in tumor epithelial cells. Collectively, the spatial transcriptomics results highlight TMB-Epi cells as central communication hubs within BLCA tissues. Their active interactions with surrounding stromal components, heightened JAK-STAT signaling, and spatial co-expression of ABRACL and ARPC3 underscore their critical contribution to tumor progression from a spatial perspective.

### 3.8 Experimental validation of ABRACL and ARPC3

**Figure 11:**
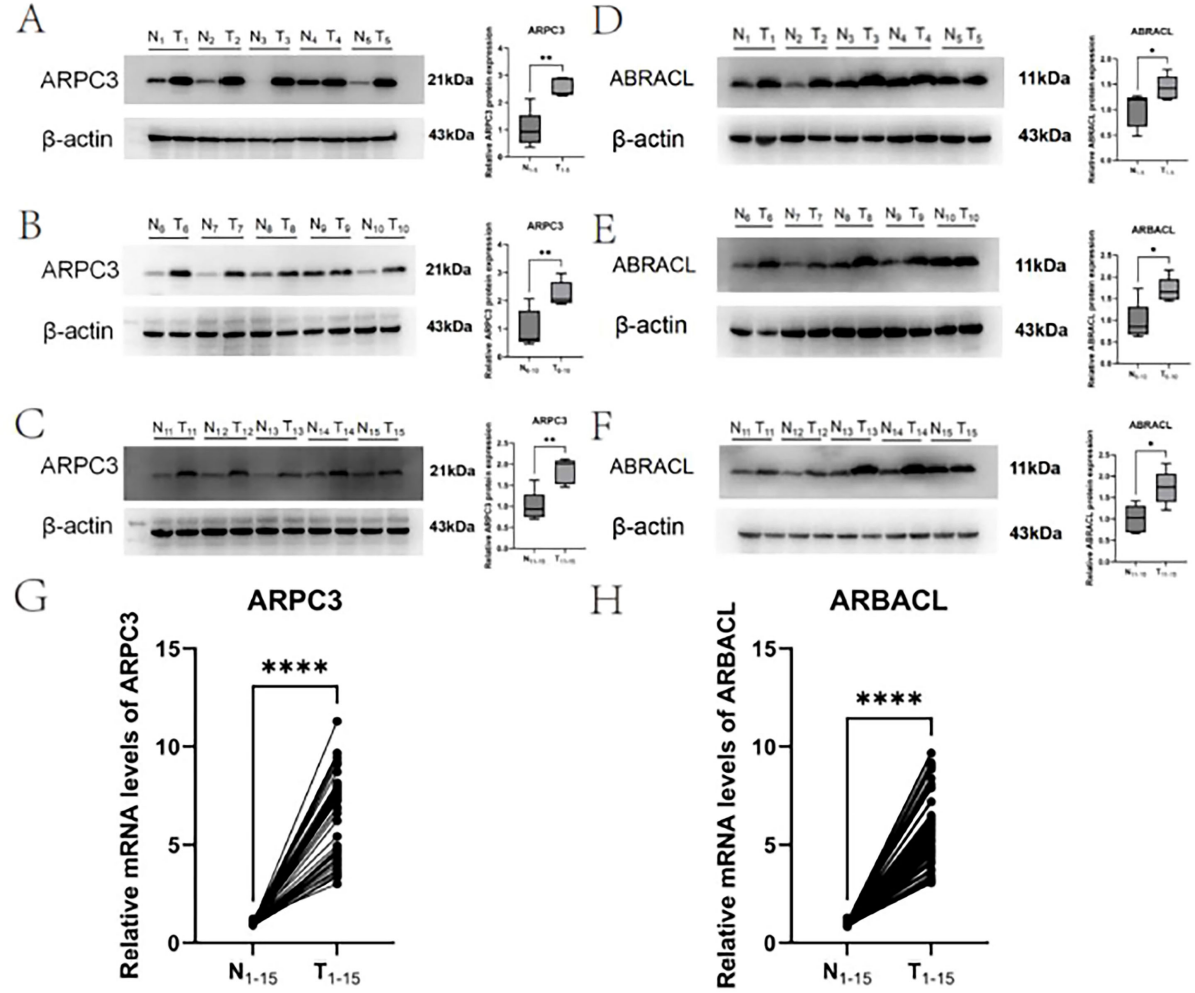
Experimental validation of ABRACL and ARPC3 expression in BLCA tissues. **(A–C)** Representative Western blot images and quantitative analysis of ARPC3 protein levels in tumor versus adjacent normal tissues. **(D–F)** Representative Western blot images and quantitative analysis of ABRACL protein levels in tumor versus adjacent normal tissues. **(G, H)** RT-qPCR results showing mRNA expression levels of ABRACL and ARPC3 in paired BLCA and adjacent tissues. **** indicates a statistical significance level of P < 0.0001.

To further validate the expression patterns of the identified hub genes, 15 pairs of BLCA tissues and matched adjacent normal tissues were analyzed for ARPC3 and ABRACL expression at both the mRNA and protein levels. RT-qPCR results showed that the mRNA expression of both genes was significantly higher in tumor tissues than in adjacent tissues (**Figures 11G, H**). Consistently, Western blot analysis demonstrated markedly stronger protein bands for ARPC3 and ABRACL in tumor samples, with statistically significant differences between tumor and normal tissues (**Figures 11A-F**). Together, these findings confirm that ARPC3 and ABRACL are significantly upregulated in BLCA, supporting their potential roles in tumor initiation and progression.

## 4 Discussion

BLCA is a highly heterogeneous malignancy characterized by substantial molecular and cellular diversity, and its development is closely associated with TMB and the TME (30, 31). Previous studies have primarily focused on TMB as a predictor of immunotherapy response; however, its mechanistic role at the cellular subpopulation and spatial levels remains unclear (32–34). In this study, we integrated self-generated WES and RNA-seq data with publicly available scRNA-seq and ST datasets to systematically elucidate the cellular composition, molecular pathways, and spatial characteristics associated with TMB in BLCA. Our findings reveal that TMB may influence tumor progression by reshaping epithelial cell states and their interactions within the TME.

Based on WES-derived TMB values and transcriptomic correlation analysis, we identified a TMB-associated epithelial subpopulation from scRNA-seq data. This subpopulation exhibited strong intercellular communication advantages, characterized by markedly enhanced ligand–receptor interactions with fibroblasts, endothelial cells, and macrophages. These findings suggest that TMB-Epi cells may represent key communication-associated epithelial states within the epithelial–stromal interaction network, potentially contributing to tumor progression (35). Importantly, CellChat analysis revealed that TMB-Epi cells preferentially interact with neighboring stromal cells through signaling axes such as MDK–SDC1 and MDK–SDC4, pathways previously linked to extracellular matrix remodeling, angiogenesis, and immune evasion (36, 37). In addition, PROGENy-based pathway analysis demonstrated significant activation of JAK–STAT and NF-κB signaling in TMB-Epi cells, indicating that high TMB tumor cells may maintain persistent inflammatory signaling to promote cell survival and enhance immune resistance (38–41).

Using the RSF algorithm, we identified ABRACL and ARPC3 as key genes closely related to tumor mutational burden (TMB). Both of these genes are closely associated with the occurrence and development of bladder cancer, and have been validated in multiple datasets (including our own cohort, TCGA, GSE236932, and GSE222315) of tumor tissues (42–44). Notably, ABRACL-high samples exhibited significantly higher TMB values, suggesting its potential involvement in mutation-driven transcriptional reprogramming. Drug sensitivity analyses further revealed that high expression of ABRACL and ARPC3 corresponded to enhanced sensitivity to chemotherapeutic agents such as gemcitabine, paclitaxel, docetaxel, and cisplatin, providing a potential framework for therapy stratification.

At the spatial level, ST data validated these findings by demonstrating distinct spatial clusters across BLCA tissues. Deconvolution analysis indicated that tumor regions were predominantly composed of epithelial cells, fibroblasts, macrophages, and immune cells, exhibiting pronounced regional heterogeneity. TMB-Epi cells were strongly enriched in tumor-dense regions, correlating with transcriptionally active areas. Moreover, ABRACL and ARPC3 displayed highly concordant high expression in tumor-enriched spatial zones (BLCA-B2), supporting the notion that ABRACL and ARPC3 are spatially co-expressed and may be jointly associated with epithelial signaling activity and cytoskeletal organization. Additionally, a nomogram incorporating ABRACL, ARPC3, and clinical features demonstrated moderate predictive performance under internal validation for 1-, 3-, and 5-year OS, validated by ROC and DCA analyses, suggesting its potential utility as an exploratory prognostic tool for survival prediction in BLCA patients.

Collectively, this multi-omics study provides comprehensive insights into TMB-associated epithelial cell states and communication networks within the BLCA TME. By integrating genomic, transcriptomic, spatial, and machine-learning analyses, we highlight ABRACL and ARPC3 as candidate regulators associated with tumor progression–related signaling features and chemotherapy response.

Despite these strengths, our study has several limitations. First, the relatively small number of self-generated samples may limit the generalizability of some findings. Second, although publicly available scRNA-seq and ST datasets offer valuable validation, matched single-cell and spatial profiling from the same patient cohort would provide even stronger evidence. Finally, the mechanistic roles of ABRACL and ARPC3 require further experimental investigation, including *in vitro* functional assays and *in vivo* animal model studies. In particular, functional validation will be a critical priority and will be pursued in future studies as conditions permit.

## 5 Conclusion

In summary, by integrating WES, bulk RNA-seq, scRNA-seq, and spatial transcriptomics, this study delineates the spatial distribution and regulatory characteristics of TMB-associated epithelial cells within BLCA tissues. TMB-Epi cells function as signaling hubs, interacting with stromal components through ligand–receptor pathways such as MDK–SDC1 and exhibiting strong activation of the JAK–STAT pathway, which may contribute to tumor cell growth and immune evasion. The high expression and spatial co-localization of ABRACL and ARPC3 in tumor-enriched regions further underscore their central roles in TMB-driven epithelial remodeling. Collectively, these findings provide novel insights into the molecular heterogeneity and spatial regulatory mechanisms of BLCA and highlight potential avenues for precision therapies targeting TMB-associated epithelial subpopulations.

## Data Availability

The datasets presented in this study can be found in online repositories. The names of the repository/repositories and accession number(s) can be found in the article/**Supplementary Material**.
